# Gene signature for response prediction to immunotherapy and prognostic markers in metastatic urothelial carcinoma

**DOI:** 10.3389/fimmu.2025.1607222

**Published:** 2025-11-20

**Authors:** Peter Langfelder, En-Tni Lin, Yi-Ta Tsai, Tai-Lung Cha, Grace S. Shieh

**Affiliations:** 1 Institute of Statistical Science, Academia Sinica, Taipei, Taiwan; 2 Center for Neurobehavioral Genetics, The Jane and Terry Semel Institute for Neuroscience and Human Behavior, University of California, Los Angeles, Los Angeles, CA, United States; 3 Department of Psychiatry and Biobehavioral Sciences, David Geffen School of Medicine, University of California, Los Angeles, Los Angeles, CA, United States; 4 Graduate Institute of Life Sciences, National Defense Medical Center, Taipei, Taiwan; 5 National Institute of Cancer Research, National Health Research Institutes, Miaoli, Taiwan; 6 Bioinformatics Program, Taiwan International Graduate Program, Academia Sinica, Taipei, Taiwan; 7 Data Science Degree Program, Academia Sinica and National Taiwan University, Taipei, Taiwan; 8 Genome and Systems Biology Degree Program, Academia Sinica and National Taiwan University, Taipei, Taiwan

**Keywords:** biomarker, cancer, immunotherapy, machine learning, regression, prediction

## Abstract

To date, immune checkpoint inhibitors (ICIs) have emerged as a leading treatment for metastatic cancer, significantly improving patient survival while causing relatively few side effects. However, the objective response rate for ICIs remains low approximately 30% in urothelial carcinoma (UC), underscoring the urgent need for predictive response biomarkers. Several state-of-the-art signatures have been revealed in top-tier journals, highlighting the importance of this field. As the number of genes (~20,000) far exceeds the sample sizes of typical training sets (generally ≤ 300), we first developed feature selection procedures to reduce the number of features to a few hundred. We then trained multiple machine learning classifiers using the selected genes and the IMvigor210 dataset, which includes RNA-seq and clinical data from ~298 patients with metastatic UC (mUC). Notably, our predictor LogitDA, using the identified 49-gene signature, achieved a prediction AUC of 0.75 in an independent dataset, PCD4989g(mUC). Moreover, our signature outperformed six state-of-the-art signatures, PD-L1 IHC, and five tumor microenvironment signatures, including IFN-γ, T-effector, and T-cell exhaustion signatures. When we integrated each of the six known signatures with our own, our signature still surpassed the integrated ones in terms of prediction AUC and accuracy in the PCD4989g(mUC) dataset. From our signature, we identified key prognostic biomarkers, with the top five markers LYRM1, RFC4, CENPL, SPAG5, and CACYBP (Benjamini-Hochberg adjusted P < 0.0025) in the IMvigor210 dataset. Finally, we performed pathway analyses using Reactome (MSigDB) and KEGG, to reveal some immune-related pathways enriched such as MHC class II antigen presentation.

## Introduction

Metastasis accounts for nearly 90% of cancer-related deaths and remains a major challenge in effective cancer treatment. Immune checkpoint inhibitors (ICIs) have improved outcomes in several metastatic cancers. In metastatic urothelial carcinoma (mUC) (1–3), the PD-L1 inhibitor atezolizumab has shown durable clinical efficacy (4). Atezolizumab, a humanized monoclonal antibody, binds PD-L1 and blocks its interaction with PD-1 and B7.1, thereby restoring tumor-specific T-cell immunity. However, only about 20% of mUC patients achieve objective responses (5, 6), highlighting the need for reliable biomarkers to predict treatment benefit before therapy initiation (7).

Banchereau et al. reported that tumor mutation burden (TMB) and PD-L1 expression had limited predictive power across the IMvigor210 (mUC), POPLAR, and IMmotion150 (RCC) cohorts, whereas RNA-seq-based models captured their effects more accurately (6). They further demonstrated that cancer-specific models outperform pan-cancer approaches. Guided by this principle, we developed an mUC-specific transcriptomic signature predictive of response to atezolizumab, aiming to stratify patients most likely to benefit from ICI therapy.

Data-driven machine learning (ML) models often fail to generalize response predictions to independent datasets (8). To address this problem, we applied transfer learning to enhance feature selection and classifier training. Because the number of genes far exceeds the number of ICI-treated samples, effective feature reduction is critical. We incorporated unsupervised domain adaptation (DA) to reduce ~17,000 genes to a few hundred, prioritizing those with similar statistical distributions across training and test datasets (9–11). This ensured that selected features retained predictive power across domains.

**Figure 1:**
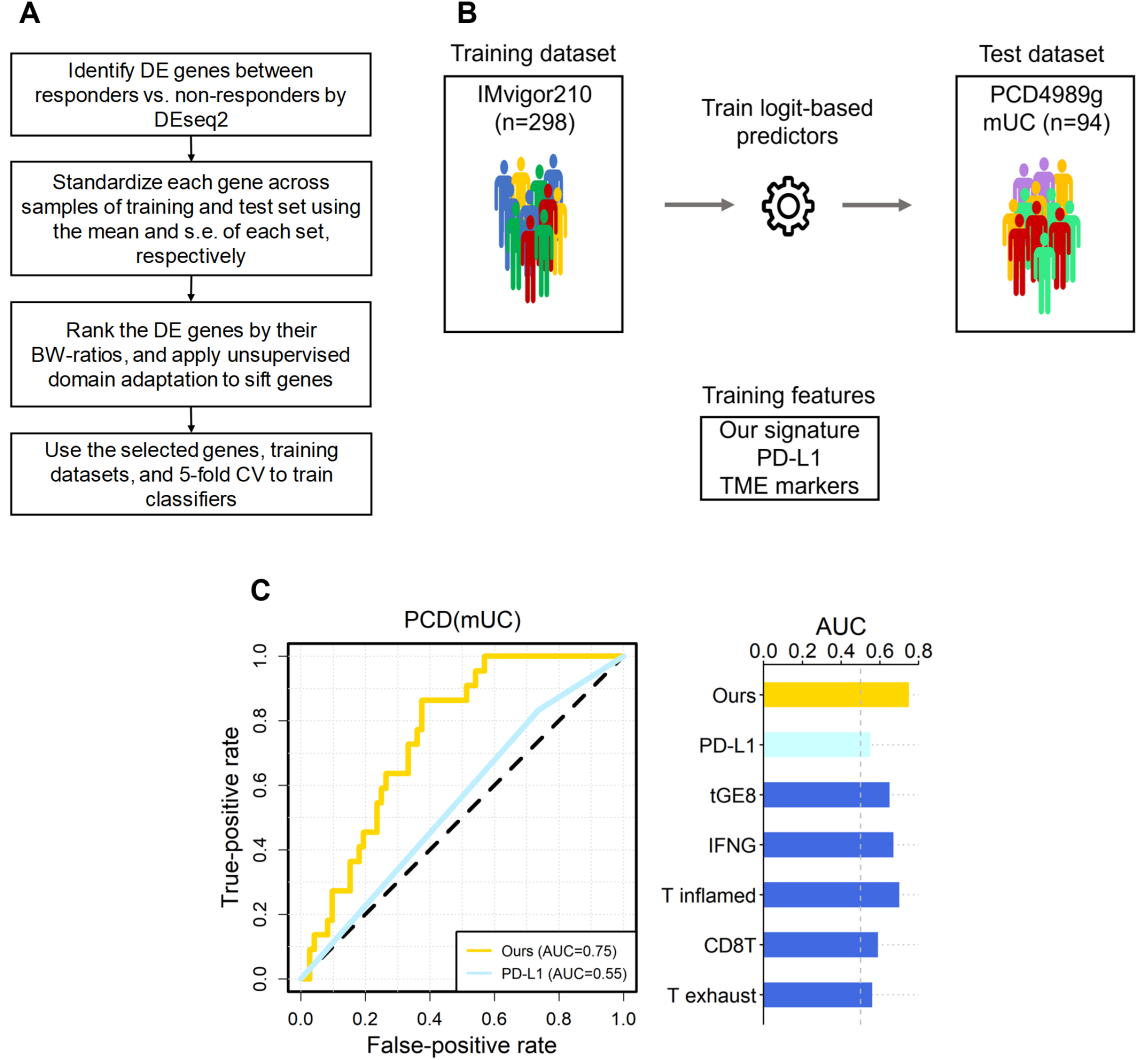
The overall scheme, and predictive performance of our signature versus the six known ones in independent mUC dataset. **(A)** The flowchart of our scheme, where the DE genes of IMvigor210 were selected by FDR < 0.10. **(B)** The scheme of training and test of logit-based predictors with our signature and the six known ones. The datasets and signatures used to train and testing the logit model, and the number of samples in each dataset are shown. **(C)** The area under the receiver operating characteristic curve (AUC) for PCD(mUC) is displayed. The random expectation (AUC = 0.5) is shown in dotted lines.

Following feature selection, we performed cross-validation of four classifiers, logit, lasso, support vector machine (SVM), and random forest, using the IMvigor210 and IMmotion150 datasets to identify the most consistent predictor. As illustrated in **Figure 1A**, LogitDA, a logistic regression-based model, consistently achieved the highest prediction accuracy across both cohorts. Consequently, LogitDA and its derived gene signature were selected for independent validation in the PCD4989g(mUC) dataset.

We further compared our 49-gene mUC signature with six established immunotherapy response predictors, including PD-L1 IHC and five tumor microenvironment (TME)-associated gene signatures: the IFN-γ signature (1), T-cell dysfunction signature (12), T-effector (tGE8) signature (2), T-cell–inflamed GEP (13), TIDE T-exhaust signature (12), and CD8T signature (14). The IFN-γ and T-cell-inflamed GEP each comprise 18 genes linked to antigen presentation, chemokine signaling, and adaptive immune resistance, and have shown pan-cancer predictive value for anti-PD-(L)1 therapy. The tGE8 signature, composed of *IFNG*, *CXCL9*, *CD8A*, *GZMA*, *GZMB*, *CXCL10*, *PRF1*, and *TBX21*, correlates with PD-L1 expression and is elevated in responders (2, 15). The TIDE model identified 50 genes associated with T-cell exhaustion (12), while the CD8T signature (*CD8A*, *CD8B*, *GZMA*, *GZMB*, *PRF1*) reflects CD8^+^ T-cell activity (14).

We found that our 49-gene signature for mUC outperformed all six known signatures in terms of prediction AUC and accuracy in an independent test dataset PCD4989g(mUC). We then evaluated whether integrating each of these six signatures with our own could improve predictive performance. However, we found that our signature still outperformed the integrated signatures in response prediction in mUC. We further showed that combining our signature and tumor mutation burden (TMB), a widely recognized genomic biomarker of ICI response, significantly improved prediction performance for atezolizumab-treated patients in the IMvigor210 cohort, compared to using TMB alone. Finally, we identified several prognostic markers within our gene signature that were able to stratify overall survival in patients with mUC. Taken together, our method, LogitDA, identified a robust gene signature for mUC that not only outperformed well-established biomarkers in response prediction, but also complemented them to improve the accuracy of immunotherapy outcome prediction.

## Materials and methods

### Data sets

We obtained gene expression profiles and clinical data of patients with mUC and renal cell carcinoma (RCC), respectively, from previously published studies (2, 6). These datasets include clinical response information for 298 patients enrolled in a single-arm phase II clinical trial evaluating atezolizumab as a first-line treatment for primarily mUC patients (IMvigor210) (16), and 77 RCC patients from a randomized phase II trial of atezolizumab vs. atezolizumab+bevacizumab vs. sunitinib in front line RCC (IMmotion150, NCT01984242) (17). To see which of the four classifiers studied can make consistent response prediction to atezolizumab, we used the IMmotion150 and the IMvigor210 datasets as the training datasets in the cross-validation study.

In addition, we obtained data from a phase I clinical trial of atezolizumab (PCD4989g, NCT01375842) (18) through a data request to Genentech (South San Francisco, USA). This dataset includes whole-transcriptome profiles and clinical information from 94 patients with mUC and was used as an independent test dataset. Details regarding the availability of raw data are provided in the Data Availability section. Each dataset contains over 30,000 transcripts with corresponding expression values in raw counts.

Clinical responses in the IMvigor210, PCD4989g, and IMmotion150 datasets were assessed according to the Response Evaluation Criteria in Solid Tumors version 1.1 (RECIST 1.1) (19), and were classified as complete response (CR), partial response (PR), stable disease (SD), or progressive disease (PD). For downstream analyses, patients were grouped into responders (CR/PR) and non-responders (SD/PD), respectively.

Among the 348 patients in the IMvigor210 trial, clinical response data were available for 298 patients, the majority of whom were diagnosed with mUC, although some also had liver, lung, or other cancers. In total, the IMvigor210 (IMmotion150) cohort included 298 (77) patients, with 68 (15) responders and 230 (62) non-responders. The PCD4989g (mUC) test dataset included 94 patients, with 22 responders and 72 non-responders.

### RNA-seq data processing and normalization

Whole transcriptomic profiles of IMvigor210 (PCD4989g) were downloaded in raw read (FASTQ) format; the reads were aligned and quantified to gene-level counts by kallisto (20). After applying DEseq2 to identify differentially expressed (DE) genes, we normalized the counts to log_2_ (TPM + 1) (21) for each sample of training and test sets.

### Standardization of training and test data

To reduce the influence of systematic technical differences between training and test data sets, we applied independent standardization (denoted as ST) to IMvigor210 and PCD4989g (mUC), that scaled each gene to mean 0 and variance 1 separately in training and test data, given the proportions of the two outcomes CR/PR and SD/PD are similar between the two datasets.

### Feature selection

For feature selection, we have implemented the following procedures. (1) We identified differentially expressed (DE) genes of responders versus non-responders by DEseq2 (22), excluding non-informative genes (HIST and LOC). (2) Next, we calculated the ratio of between-group to within-group sums of squares (BW-ratio) (23) for the DE genes, where the two groups refer to responders and non-responders. and (3) Finally, we applied unsupervised domain adaptation (DA) (10) to sift genes which passed step (2). Details of the feature selection procedures are in **Supplementary Materials**.

### Training the four classifiers by 5-fold CV

After completing the feature selection procedures, we used the IMvigor210 dataset to train the parameters of four classifiers, logistic regression, lasso regression, random forest (RF), and support vector machine with the RBF kernel (SVM(RBF)), via 5-fold cross-validation. For LogitDA, the parameters *p* of the top-*p* genes, α_DA_ of DA and the penalty constant *λ* of logit regression were optimized by grid search with 5-fold CV and 100 repeats to result in the associated predictors. Specifically, *p* is evaluated from the union of [15(5)200]) and [210(10)500], where the former denotes the set of numbers from 15 with step size 5 to 200 for IMvigor210, and α_DA_ in [0.2, 0.8] (step size = 0.1). Further details of the training procedures for each classifier are provided in the **Supplementary Materials**.

### Statistical analysis and tools

We used the DEseq2 and dgof packages in R software to identify DE genes between responders and non-responders. A log-rank test was applied to reveal genes that can separate patients into favorable or poor OS within IMvigor210. For the remaining analyses, including logistic ridge regression, support vector machine, random forest classification, and visualization (e.g., volcano plots), we used R software.

## Results

### Within-study cross validations demonstrated that LogitDA consistently predicted responses in atezolizumab-treated patients

After applying the feature selection procedures, we performed 5-fold cross-validation (CV) with 100 repeats on the IMvigor210 training dataset using four classifiers, LogitDA, lasso regression, random forest, and support vector machine with the RBF kernel (SVM(RBF)). To determine which classifier provided the most robust predictions across cancer types in atezolizumab-treated patients, we also performed a 5-fold CV study using the IMmotion150 (mRCC) dataset.

In the IMvigor210 dataset, lasso and LogitDA achieved the highest CV AUCs (accuracy) of 0.78 and 0.77 (0.71 and 0.73), respectively, followed by SVM(RBF) and random forest. AUC (area under the receiver operating characteristic curve) was used as the primary metric for classifier performance evaluation. In the IMmotion150 cohort, LogitDA and SVM(RBF) were the top two predictors, each attaining a cross-validated AUC of 0.95.

Detailed CV results of both datasets, including AUC, accuracy, true positives (TPs), true negatives (TNs), and other metrics, are provided in **Supplementary Table S1**. Based on the results from both datasets, we selected LogitDA and the 49-gene signature it identified for further investigation.

### Our signature outperformed the six well-known signatures in ICI-treated patients with mUC

Let IMvigor-PCD(mUC) denote the setting in which a classifier is trained on the IMvigor210 dataset and its prediction performance evaluated on the PCD4989g(mUC) dataset. In this section, using IMvigor-PCD(mUC), we compared the performance of our gene signature with six state-of-the-art signatures, PD-L1 (IHC), IFN-γ (1), tGE8 (2), T exhaust (12), T inflamed (13), and CD8T (14), as introduced in the *Introduction*. We first conducted 5-fold cross-validation with 100 repeats for each signature using logistic regression combined with our optimization algorithm to derive the corresponding predictors. In the IMvigor210 dataset, our signature achieved the highest cross-validated AUC of 0.77, followed by IFN-γ (0.70), T inflamed (0.69), tGE8 (0.68), and T exhaust (0.62); both CD8T and PD-L1 yielded cross-validated AUCs below 0.60.

Next, we applied the trained predictors to the independent test set, PCD(mUC). Our transcriptomic signature outperformed the six established signatures, achieving the highest prediction AUC of 0.75, followed by T inflamed (0.70), IFN-γ (0.67), tGE8 (0.65), and the remaining signatures (0.59 and lower), as shown in **Table 1**. In terms of prediction accuracy, our signature also ranked highest at 0.71, followed by IFN-γ (0.67), with the others performing at 0.64 or lower. Since the PCD(mUC) dataset does not include tumor mutation burden (TMB) information, we were unable to evaluate the predictive power of TMB. Detailed metrics, including prediction AUC, accuracy, true positives (TPs), true negatives (TNs), false positives (FPs), and false negatives (FNs), are provided in **Table 1**. An overview of the training and testing scheme for LogitDA and logistic regression-based predictors, along with their corresponding prediction AUCs in PCD(mUC), is illustrated in **Figure 1**.

**Table 1:** Our signature for mUC outperformed the six known ones in response prediction to Atezolizumab using IMvigor210-PCD(mUC).

Signatures	Parameter	CV result	Prediction result						
	*λ* ^a^	AUC (SE)	AUC	accuracy	F1-score	TPs	TNs	FPs	FNs
Ours (49)^b^	0.14	0.77 (0.00^c^)	0.75	0.71	0.47	12	55	17	10
PD-L1^d^	1.07	0.57 (0.001)	0.55	0.40	0.40	15	15	42	3
tGE8 (8)	0.05	0.68 (0.01)	0.65	0.61	0.39	12	45	27	10
IFN-γ (18)^e^	0.05	0.70 (0.01)	0.67	0.67	0.46	13	50	22	9
T inflamed (17)^e^	0.04	0.69 (0.02)	0.70	0.64	0.43	13	47	25	9
CD8T (5)	1.04	0.58 (0.01)	0.59	0.61	0.39	12	45	27	10
T exhaust (50)	0.15	0.62 (0.02)	0.56	0.55	0.28	8	44	28	14

a *λ* is the penalty constant of logistic ridge regression.

b LogitDA with the optimized α_DA_ = 0.2 and *λ=* 0. 15 resulted in the 49-gene model.

c SE equals to “0.00” after rounded to the 3^rd^ digit.

d After excluding samples with PD-L1 (IHC) missing, the sample size of IMvigor210 and PCD(mUC) were 297 and 75, respectively.

e In the IFN-γ and T inflamed signatures, HLA-E was missing in IMvigor-PCD(mUC).

### Our signature outperformed the six integrated signatures in response prediction to atezolizumab in mUC

After demonstrating that our signature outperformed the six state-of-the-art signatures individually, we investigated whether combining any of these signatures with ours could further improve predictive performance. As shown in **Table 2**, our signature achieved the highest prediction AUC (0.75) and accuracy (0.71) in the mUC test set. Specifically, integrating PD-L1 (IHC) and the CD8T signature with ours resulted in prediction AUCs of 0.69 and 0.73, and accuracies of 0.65 and 0.68, respectively. Interestingly, combining PD-L1 with our signature (denoted as PD-L1+ours) led to a reduction in both AUC and accuracy compared to using our signature alone. Nevertheless, the combination CD8T+ours achieved the highest number of true positives (13) among all integrated signatures, while our signature yielded 12 true positives prediction.

**Table 2:** Our signature surpassed the integrated signatures in response prediction to Atezolizumab using IMvigor210-PCD(mUC).

Signatures	Parameter		Cross-validation result	Prediction result						
	*p*	*λ* ^a^	AUC (SE)	AUC	accuracy	F1- score	TPs	TNs	FPs	FNs
Ours (49)^b^	49	0.14	0.77 (0.00^c^)	0.75	0.71	0.47	12	55	17	10
PD-L1+ours^d^	50	0.17	0.78 (0.01)	0.69	0.65	0.35	7	42	15	11
tGE8+ours^e^	56	0.17	0.78 (0.01)	0.71	0.66	0.41	11	51	21	11
IFN-γ+ours^f^	66	0.19	0.77 (0.01)	0.71	0.66	0.41	11	51	21	11
T inflamed+ours^f^	65	0.18	0.77 (0.01)	0.72	0.66	0.43	12	50	22	10
CD8T+ours	54	0.18	0.77 (0.01)	0.73	0.68	0.46	13	51	21	9
T exhaust+ours	99	0.27	0.75 (0.01)	0.71	0.71	0.34	7	60	12	15

a *λ* is the penalty constant of logistic ridge regression.

b LogitDA with the optimized α_DA_ = 0.2 and *λ=* 0. 15 resulted in the 49-gene model.

c SE equaled to “0.00” after rounded to the 3^rd^ digit.

d After excluding samples with PD-L1 (IHC) missing, the sample size of IMvigor210 and PCD(mUC) were 297 and 75, respectively.

e The tGE8 signature having *CXCL9* overlapped with our signature.

f Of the IFN-γ and Cristescu signaturees, *CXCL9* overlapped with our signature, and *HLA-E* was missing in the IMvigor210-PCD(mUC) datasets.

### Differentially-expressed *GABRA3*, *MAST1*, *CXCL*9, *NUF2*, and *LURAP1* were associated with response to Atezolizumab in patients with mUC

From the volcano plot of our 49-gene signature for IMvigor210-PCD(mUC) (**Supplementary Figure S1**), we identified several genes significantly associated with response to atezolizumab in patients with mUC. We found that *GABRA3*, *MAST1*, *CXCL9*, and *NUF2* were the most over-expressed genes, with log_2_ fold changes of 1.26, 1.24, 1.12, and 0.90, Benjamini-Hochberg adjusted *p*-values of 6.0×10^-4^, 8.8×10^-7^, 0.0025, and 1.4×10^-6^, respectively). Conversely, *LURAP1* was the most underexpressed gene with a log_2_ fold change of -0.88 and adjusted *P =*1.5×10^-5^.

We checked these four over-expressed genes against existing literature, and found the following. Gene *GABRA3* has been associated with TMB and shown to promote antitumor immunity in hepatocellular carcinoma based on multi-omics analysis (24). Recent studies have also reported that manipulating GABAergic signaling could limit anti-tumor immunity (25, 26). Since response to immune checkpoint blockade (ICB), such as PD-L1 inhibition, is known to correlate with the extent of tumor immune infiltration, we further investigated immune-related functions of the identified genes. Several reports indicate that *CXCL9* is associated with immune cell infiltration (27) and is required for effective antitumor responses following ICB treatment (28).

In addition, Zheng B et al. demonstrated that *NUF2* positively correlates with tumor-infiltrating immune cells, including CD8^+^ T cells and dendritic cells, in clear renal cell carcinoma (29). Notably, our finding that under expression of *LURAP1* is associated with better response to atezolizumab is consistent with previous evidence from TCGA bladder cancer data, where hypermethylation of five CpG sites in *LURAP1* (resulting in reduced expression) was linked to improved overall survival (30).

### Prognostic biomarkers of overall survival identified for mUC

**Figure 2:**
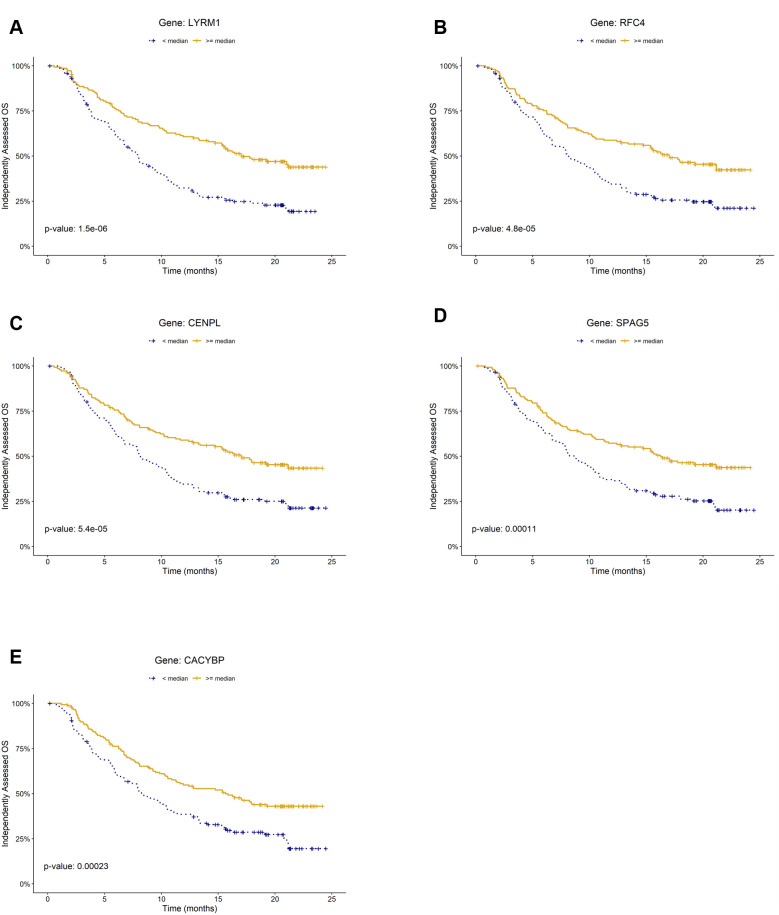
Kaplan–Meier plots of prognostic biomarkers for overall survival (OS) in the mUC cohort (IMvigor210). Panels **(A–E)** show survival curves stratified by gene expression levels of **(A)***LYRM*, **(B)***RFC4*, **(C)***CENPL*, **(D)***SPAG5*, and **(E)***CACYBP*, respectively. For each gene, patients were classified into high (≥ median) and low (< median) expression groups within the cohort.

To identify prognostic markers for overall survival (OS), we conducted log-rank tests on the 49 genes included in the LogitDA predictor for the IMvigor210-PCD(mUC) setting. We identified 18 genes as significant prognostic biomarkers, each with a Benjamini–Hochberg adjusted *P* value (FDR) < 0.01 (log-rank test; **Supplementary Table S2**). The top five ranked biomarkers were *LYRM1*, *RFC4*, *CENPL*, *SPAG5*, and *CACYBP*, all with adjusted *P* values < 0.0025. Kaplan–Meier survival curves for these five genes are shown in **Figures 2A–E**.

### Pathway analysis and functional roles of some signature genes

To elucidate the biological processes captured by our signature, we performed pathway analyses using Reactome (MSigDB) and KEGG. Reactome analysis revealed significant enrichment of MHC class II antigen presentation (P = 0.013) and aberrant mitotic exit regulation (P = 0.029) among the 29 upregulated genes, with marginal enrichment of adaptive chemokine receptor binding (P = 0.08) and immune system pathways (P = 0.09). The 20 downregulated genes were enriched in the cell cycle pathway (P = 0.02). KEGG analysis further identified enrichment in cell cycle, DNA replication, HTLV-1 infection, and DNA repair pathways (adjusted P = 1.9 × 10^-16^ - 0.005). Details are provided in **Supplementary Table S3**.

We next examined the functional roles of high-weight and differentially expressed signature genes. Literature evidence supports their involvement in tumor immunity and immunotherapy response. CXCL9, an IFN-γ-inducible chemokine that recruits CXCR3^+^ effector T and NK cells, is central to anti-tumor immunity (28, 31). In mUC (IMvigor210), elevated CXCL9/IFNG/GBP5 expression correlates with favorable anti-PD-L1 response (32), consistent with the IFN-γ→CXCL9 axis. *NUF2* expression positively correlates with tumor-infiltrating CD8^+^ T cells and dendritic cells in clear renal cell carcinoma, while *POLA2* has been identified as a positive biomarker for PD-L1 blockade response in mUC. LURAP1, an adaptor activating the canonical NF-κB pathway, promotes PD-L1 expression and immune evasion; its underexpression in responders aligns with enhanced ICB efficacy (33). CDCA3/5/8, implicated in immune-related pathways (34), were negatively associated with ICI response in our study, consistent with their reported roles in suppressing CD8^+^ T-cell infiltration. Additionally, *SLC6A1* and *GABRA3*, differentially expressed between responders and non-responders, participate in the GABAergic pathway, whose aberrant activation has been linked to immune suppression in the tumor microenvironment (25, 26).

### Ablation study

In this section, we evaluated the contribution of domain adaptation (DA) to the predictive performance of LogitDA. We trained logistic regression models using features selected from the first two feature selection steps, namely excluding the DA filtering step. All model parameters except for αDA​ were optimized using 5-fold cross-validation on the IMvigor210 dataset. The resulting predictor, denoted as LogitDA**–**DA (i.e., without DA), included 150 genes. When applied in the IMvigor-PCD(mUC) setting, this model yielded a prediction AUC of 0.63. In comparison, LogitDA (with DA) achieved a 12% improvement in AUC, demonstrating the benefit of incorporating DA.

## Discussion

In this study, we developed a feature selection pipeline and a machine learning–based predictor, LogitDA, for identifying a transcriptomic signature of response to PD-L1 inhibition in mUC. We demonstrated that LogitDA robustly predicted patient response to atezolizumab and identified an effective gene signature associated with treatment outcome. Using LogitDA, we further showed that the identified mUC-specific signature outperformed PD-L1 (IHC) as well as five established tumor microenvironment (TME)-associated signatures in terms of both prediction AUC and accuracy.

Furthermore, we integrated our mUC-specific signature with each of the six established immune-related signatures and found that our signature achieved the highest predictive performance, with a prediction AUC of 0.75, outperforming all integrated combinations. These findings suggest that our method is able to identify an effective transcriptomic signature from ICI-treated patients with mUC, and may provide a useful tool for stratifying patients likely to benefit from atezolizumab therapy.

In this study, we developed LogitDA, a feature selection and machine learning–based predictor, to identify a transcriptomic signature of response to PD-L1 inhibition in metastatic urothelial carcinoma (mUC). LogitDA robustly predicted response to atezolizumab and yielded an mUC-specific gene signature that outperformed PD-L1 (IHC) and five established tumor microenvironment (TME)–associated signatures in both AUC and accuracy. When integrated with these immune-related signatures, our signature achieved the highest predictive performance (AUC = 0.75). These results demonstrate that LogitDA effectively identifies clinically relevant transcriptomic predictors and may aid in stratifying mUC patients likely to benefit from atezolizumab.

TMB is a well-established genomic biomarker of response to immune checkpoint inhibitors (ICIs) across several cancer types, e.g., melanoma. Elevated TMB levels are thought to increase neoantigen load, thereby enhancing T cell infiltration and the efficacy of immunotherapy. Unfortunately, the test dataset we assessed did not comprise TMB levels. Thus, we studied the CV result of TMB alone and the combined TMB+ours signature using IMvigor210. The leave-one-out cross-validation AUCs for TMB alone and TMB+ours for mUC were 0.44 and 0.78, respectively. Importantly, we found that the combined TMB+ours signature correctly reclassified 28 non-responders previously misclassified as responders by TMB alone (R2NR), and correctly reclassified 12 responders from previously predicted non-responders by TMB alone (NR2R) for mUC, as summarized in **Supplementary Table S4**. These results suggest that integrating our signature with TMB can improve the prediction of ICI response in patients with mUC.

Using CIBERSORT (35), we deconvoluted the bulk RNA-seq data from the IMvigor210 cohort to estimate immune cell composition. Among the inferred cell types, plasma cells and M1 macrophages were significantly positively associated with patient response to atezolizumab (*P* < 0.05, FDR < 14%; Welch’s *t*-test). To further explore the immunological relevance of our signature for mUC, we compared our genes against the LM22 reference marker gene expression matrix of CIBERSORT. We found that *MAST1* and *CXCL9* were overlapping genes, and their overexpression was positively correlated with atezolizumab response (IMvigor210).

We acknowledge that the binary classification of responders (Rs) and non-responders (NRs) may limit the clinical granularity of the LogitDA predictor. To evaluate this, we compared the distributions of signature scores among CR/PR, SD, and PD groups using the Mann-Whitney test. The scores of SD were intermediate between those of CR/PR and PD. However, the difference between SD and PD was not significant (P = 0.85), whereas both CR/PR vs. SD and CR/PR vs. PD comparisons were highly significant (P < 2 × 10^-11^). These results support the appropriateness of binary classification for patient response in the IMvigor210 cohort.

Our methods can be applied to predict ICI treatment response in patients with other cancer types, provided that gene expression data and response outcomes are available for both training and test sets. Furthermore, these datasets are not overly heterogeneous (see also future research directions). Langfelder et al. demonstrated that LogitDA (36), trained on IMmotion150 (a mRCC cohort), identified a 27-gene signature that achieved a prediction AUC (accuracy) of 0.72 (0.83) in PCD4989g (mRCC) (**Supplementary Table S5**).

To evaluate the clinical generalizability of our signature, we applied it to four independent, unseen datasets GSE176307 (bladder cancer (BLCA), n = 89), GSE111636 (BLCA, n = 11), GSE91061(Melanoma, n = 49), and IMmotion150 (mRCC, n =77), yielding prediction accuracies of 61%, 73%, 67%, and 62%, respectively (**Supplementary Table S6**). These results may indicate that the signature captures key immune pathways and genes associated with ICI response. However, its predictive performance in external cohorts may depend on the similarity of their 49-gene expression profiles to those of the PCD4989g (mUC) reference set.

Given their translational potential, the 49 signature genes could be developed into a cost-effective diagnostic microarray to evaluate response to ICI therapy in patients with advanced or metastatic UC prior to treatment initiation. To assess the temporal dynamics of patient response and guide adaptive therapy, we analyzed conditional survival curves by *condSURV* package in R (Q1-Q3 expression levels; **Supplementary Figure S2**) for the top five prognostic biomarkers. Responders and non-responders began to diverge at 3–4 months, reached maximal separation at 18–21 months, and plateaued thereafter. These results indicate that biomarker discrimination peaks during the mid-term follow-up, providing the greatest clinical utility for monitoring response and informing therapy adjustment within the first 18–21 months.

Several promising gene signatures predicting ICI response have been uncovered by machine learning approaches similar to ours. Shen et al. analyzed glycolysis-related genes (37). They derived an 18-gene signature that achieved a time-dependent ROC AUC of 0.71 in IMvigor210 at 20 months. Notably, several identified genes were significantly associated with response to anti-PD-(L)1 therapy across IMvigor210 and four cohorts. Boll et al. integrated six independent cohorts, encompassing multi-omic data, immune signatures, and others (38). They derived a random forest model, reaching an AUC of 0.76 in a validation set (n = 205) combining IMvigor210 with other cohorts.

We further evaluated three recently reported ICI-response signatures (39–41) using the IMvigor210 (mUC) cohort. The NCOA3/HSP90α/EZH2/CXCL9 axis identified in colorectal cancer by Liu et al. impaired anti-PD-L1 efficacy via CXCL9 suppression; applying this signature (NCOA3, HSP90AA1, EZH2 up, CXCL9 down) for non-responders yielded an accuracy of 0.73. Ke et al. reported PD-1/CD69 co-expression in effector-memory CD8^+^ T cells as predictive of TLS formation and ICI benefit; classifying mUC patients with both PDCD1 and CD69 above the median as responders achieved 0.57 accuracy. Xu et al. defined a disulfidptosis-related signature (DFRS) associated with poor survival in several cancers but not in bladder cancer (P = 0.177, Fig. 4 (41)); similarly, DFRS failed to stratify IMvigor210 patients (P = 0.092, log-rank) and achieved 0.54 accuracy when high-DFRS cases were predicted as responders.

Recent studies on immunotherapy response prediction in urothelial carcinoma (UC) have explored biomarkers across genetic, proteomic, and transcriptomic levels from tumor, blood, and urine samples. Major research directions include: (1) Combinatorial biomarkers and computational models, particularly ML-based approaches. Yoshida et al. reported that co-expression of LAG-3 and FGL1 was linked to poor PD-(L)1 response and survival, suggesting potential benefit from combined anti-LAG-3/PD-(L)1 therapy (42). (2) DNA damage repair (DDR) alterations, such as mutations in *RB1*, *ATM*, *BRCA1/2*, and *ERCC2*, which predict ICI benefit or post-chemotherapy responsiveness (43, 44). In a phase II trial, durvalumab plus olaparib failed to improve PFS in unselected mUC patients (n = 154), but higher response rates were observed in those with homologous recombination repair mutations (45). (3) Integration of bulk and single-cell RNA-seq, and increasingly multi-omics data, to identify ICI-associated gene signatures (46–48).

The current optimization method of LogitDA focuses primarily on maximizing cross-validated AUC within the training set. In future work, we plan to apply LogitDA to other cancer types, where the training and test datasets may exhibit greater heterogeneity than those used in this study. Therefore, an important research direction will be to define and quantify the degree of heterogeneity or “distance” between training and test datasets, and to incorporate this measure into stricter criteria for transferring features across studies. Another direction of future investigation is the development of data augmentation techniques to address the class imbalance problem, as the number of responders to ICI treatments is much lower than that of non-responders. Finally, we aim to integrate single-cell RNA-seq with bulk RNA-seq data in mUC; this may elucidate which cell type proportions, and which genes in which cell types are involved in response to ICIs such as atezolizumab.

## Data Availability

The data analyzed in this study is subject to the following licenses/restrictions: Data for the clinical trial IMvigor210 is available in European Genome-Phenome Archive (EGA) under accession number EGAS00001002556. The dataset is also included in the R data package IMvigor210CoreBiologies for the R, accessible at http://research-pub.gene.com/IMvigor210CoreBiologies/. Data for the clinical trials IMmotion150 and PCD4989g are available under restricted access in European Genome-Phenome Archive with the reference number EGAS00001004343. Raw and clinical data of PCD4989g were accessed via Genentech’s internal Strand pipeline (South San Francisco, USA), and downloaded using pEGA3 (https://github.com/EGA-archive/ega-download-client). Requests to access these datasets should be directed to https://ega-archive.org/studies/EGAS00001002556; https://ega-archive.org/studies/EGAS00001004343.
