# Supplementary material for: Gene signature for response prediction to immunotherapy and prognostic markers in metastatic urothelial carcinoma
Source: Front Immunol. 2025 Nov 20;16:1607222. doi: 10.3389/fimmu.2025.1607222 (PMC12675356; doi:10.3389/fimmu.2025.1607222)
Supplement: Supplementary file 1 [file Image1.pdf]

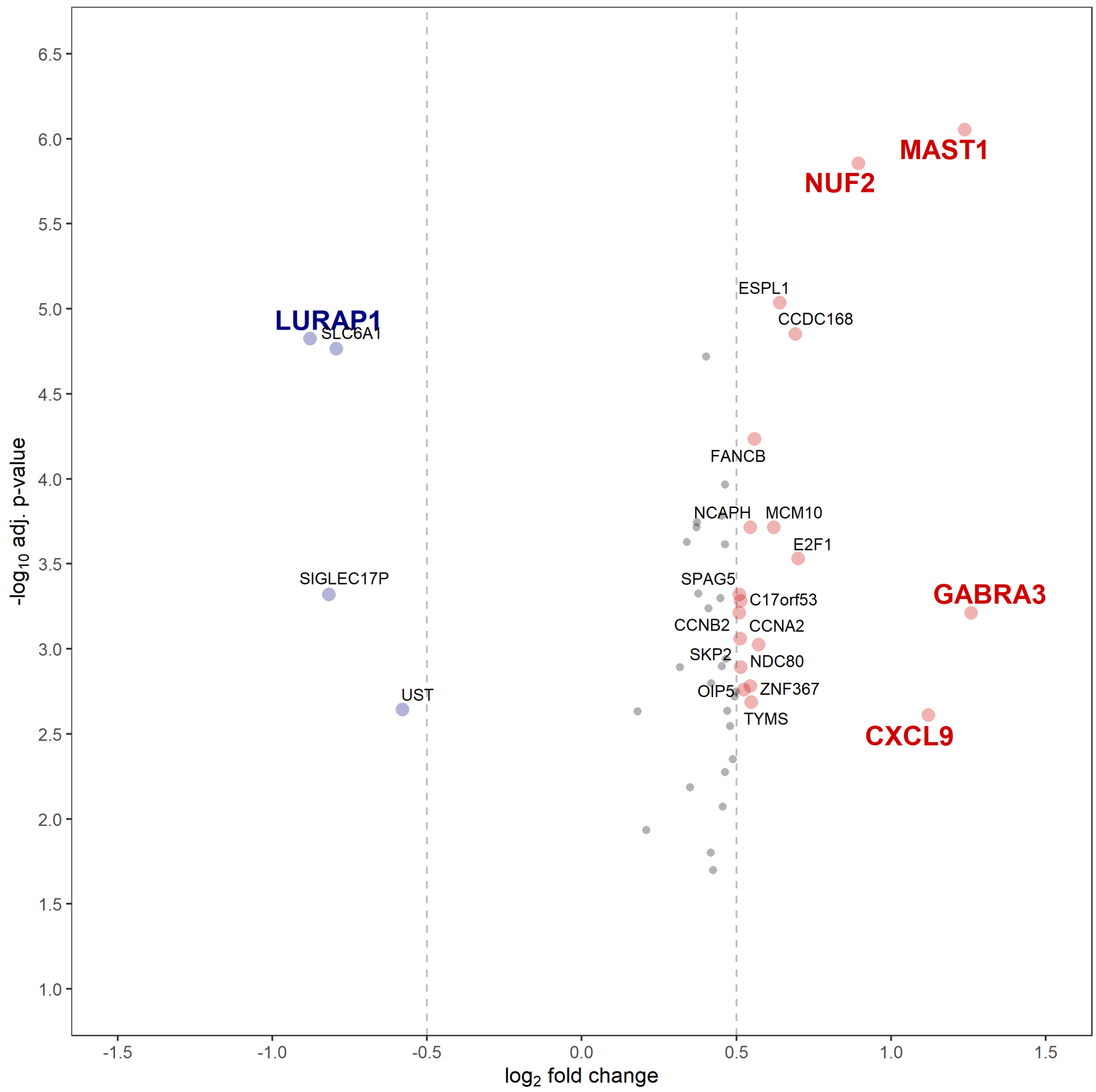

Supplementary Figure S1. Volcano plot of the 49 genes in the LogitDA model predictor of IMvigor210-PCD(mUC). The plot displays five genes differentially expressed between responders (CR/PR) versus non-responders (SD/PD), identified after Benjamini-Hochberg correction (adjusted $P < 0.005$) and an absolute $\log_2$ fold change (FC) ≥ 0.85.
