# Supplementary material for: Gene signature for response prediction to immunotherapy and prognostic markers in metastatic urothelial carcinoma
Source: Front Immunol. 2025 Nov 20;16:1607222. doi: 10.3389/fimmu.2025.1607222 (PMC12675356; doi:10.3389/fimmu.2025.1607222)
Supplement: Supplementary file 2 [file Image2.pdf]

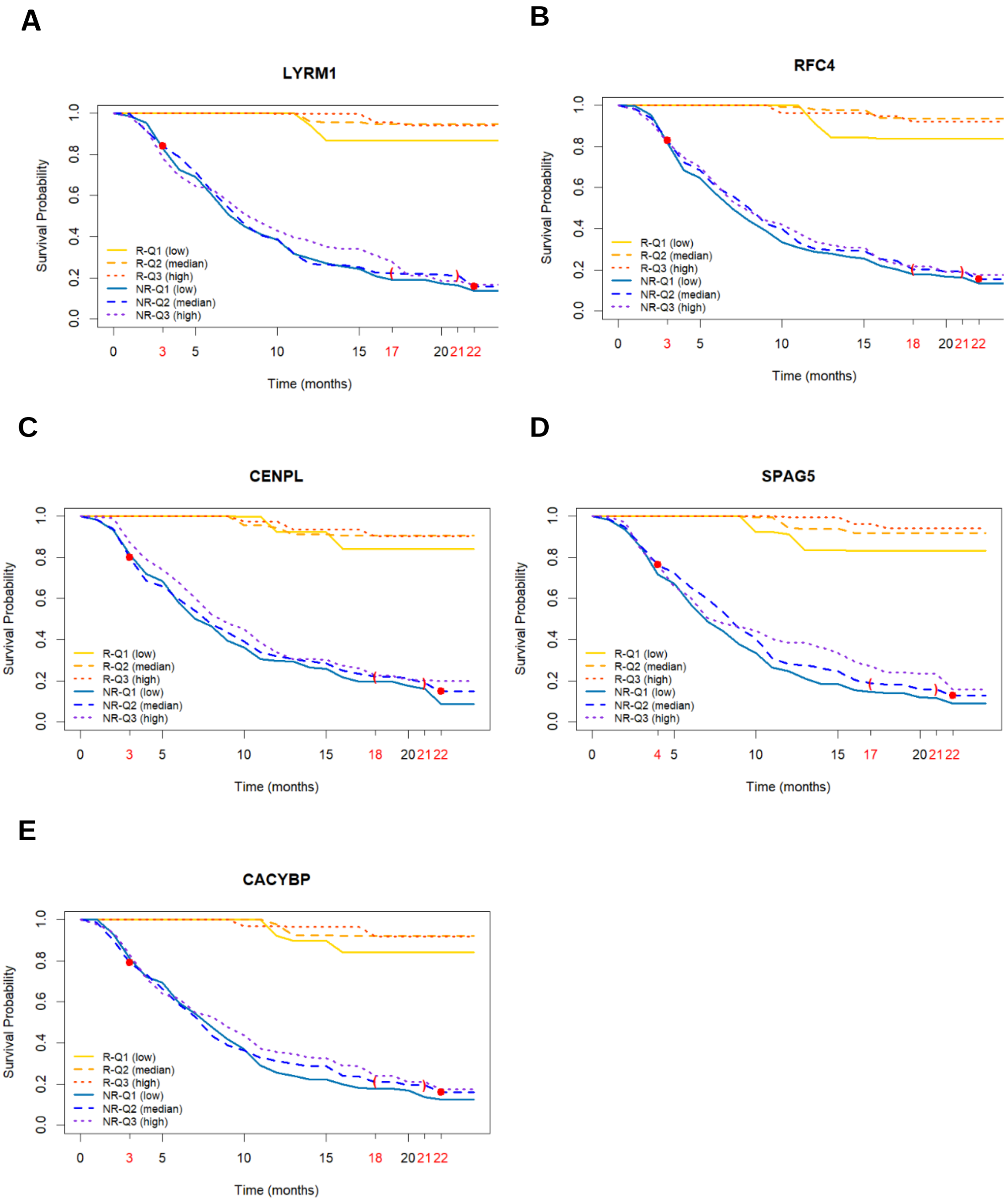

Supplementary Figure S2. Conditional survival curves illustrating time-dependent survival probabilities conditional on expression of the top-five prognostic markers in the IMvigor210 cohort. Panels (A-E) show results for *LYRM1*, *RFC4*, *CENPL*, *SPAG5*, and *CACYBP*, respectively. Each curve represents the estimated conditional survival probability over time at the genes' Q1, Q2, and Q3 expression levels for responders (R) and non-responders (NR), respectively.
