# Supplementary material for: Gene signature for response prediction to immunotherapy and prognostic markers in metastatic urothelial carcinoma
Source: Front Immunol. 2025 Nov 20;16:1607222. doi: 10.3389/fimmu.2025.1607222 (PMC12675356; doi:10.3389/fimmu.2025.1607222)
Supplement: Supplementary file 3 [file Table1.docx]

**Supplementary Table S1. Cross validation performances of four classifiers in mUC and RCC.**

| Training set | Method | Parameter  tuning | | Cross  validation  result | | | | | | | |
| --- | --- | --- | --- | --- | --- | --- | --- | --- | --- | --- | --- |
|  |  | p^a^ | α_DA_ | *λ*^b^ | AUC (SE) | accuracy | F1  score | TPs | TNs | FPs | FNs |
|  |  | nGenes^a^ |  |  |  |  |  |  |  |  |  |
| IMvigor210 | Logistic  Ridge  Regression | 49 | 0.2 | 0.14 | 0.77  (0.00^c^) | 0.73 | 0.52 | 44 | 172 | 58 | 24 |
|  |  | **150** |  |  |  |  |  |  |  |  |  |
| IMmotion150 |  | 27 | 0.2 | 0.05 | 0.95  (0.00^c^) | 0.89 | 0.76 | 14 | 55 | 7 | 1 |
|  |  | **50** |  |  |  |  |  |  |  |  |  |
| IMvigor210 | Lasso  Regression | 48 | 0.2 | 0.03 | 0.78  (0.00^c^) | 0.71 | 0.52 | 46 | 167 | 63 | 22 |
|  |  | **145** |  |  |  |  |  |  |  |  |  |
| IMmotion150 |  | 14 | 0.2 | 0.01 | 0.93  (0.00^c^) | 0.86 | 0.70 | 12 | 54 | 8 | 3 |
|  |  | **25** |  |  |  |  |  |  |  |  |  |
| IMvigor210 | Random Forest | 40 | 0.3 | No. tree = 1000 | 0.67  (0.00^c^) | 0.80 | 0.33 | 15 | 224 | 6 | 53 |
|  |  | **150** |  |  |  |  |  |  |  |  |  |
| IMmotion150 |  | 22 | 0.2 |  | 0.83  (0.00^c^) | 0.82 | 0.29 | 3 | 60 | 2 | 12 |
|  |  | **40** |  |  |  |  |  |  |  |  |  |
| IMvigor210 | SVM radial | 49 | 0.2 | C=1.06  γ=0.004 | 0.76  (0.00^c^) | 0.80 | 0.33 | 15 | 223 | 7 | 53 |
|  |  | **155** |  |  |  |  |  |  |  |  |  |
| IMmotion150 |  | 27 | 0.2 | C=5.20  γ=0.01 | 0.95  (0.00^c^) | 0.88 | 0.69 | 10 | 58 | 4 | 5 |
|  |  | **50** |  |  |  |  |  |  |  |  |  |

^a^p denotes the top-p genes sifted by the feature selection procedure, and nGenes denotes the number of genes passed the first two feature selection procedures.

^b^*λ* is the penalty constant of logistic ridge and lasso regression

^c^SE equals to “0.00” after rounded to the 3^rd^ digit.
