# Supplementary material for: Gene signature for response prediction to immunotherapy and prognostic markers in metastatic urothelial carcinoma
Source: Front Immunol. 2025 Nov 20;16:1607222. doi: 10.3389/fimmu.2025.1607222 (PMC12675356; doi:10.3389/fimmu.2025.1607222)
Supplement: Supplementary file 7 [file Table5.docx]

**Supplementary Table S5. The prediction result of LogitDA trained by IMmotion150 and tested using PCD4989g(mRCC).**

| Signature  (No. of genes) | Parameter | CV  result | Prediction result | | | | | | |
| --- | --- | --- | --- | --- | --- | --- | --- | --- | --- |
|  | *λ*^a^ | AUC  (SE) | AUC | accuracy | F1-score | TPs | TNs | FPs | FNs |
| Ours (27)^b^ | 0.05 | 0.95  (0.00^c^) | 0.72 | 0.83 | 0.44 | 4 | 44 | 6 | 4 |

*^a^*$\lambda$ is the penalty constant of logistic ridge regression

^b^LogitDA with the optimized α_DA_ = 0.2 and *λ=* 0.14 resulted in the 32-gene model.

^c^SE equals to “0.00” after rounded to the 3^rd^ digit.
