# Supplementary material for: Gene signature for response prediction to immunotherapy and prognostic markers in metastatic urothelial carcinoma
Source: Front Immunol. 2025 Nov 20;16:1607222. doi: 10.3389/fimmu.2025.1607222 (PMC12675356; doi:10.3389/fimmu.2025.1607222)
Supplement: Supplementary file 8 [file Table6.docx]

| Dataset | No. of Samples | AUC | Accuracy |
| --- | --- | --- | --- |
| BLCA Kim  (GSE176307) | 89 | 0.61 | 0.61 |
| BLCA Monero  (GSE111636) | 11 | 0.83 | 0.73 |
| mRCC  ( IMmotion150) | 77 | 0.55 | 0.62 |
| Melanoma Riaz  (GSE91061) | 49 | 0.55 | 0.67 |

**Supplementary Table S6. Prediction results for various datasets using LogitDA with the 49-gene signature in mUC.**
