# Supplementary material for: Gene signature for response prediction to immunotherapy and prognostic markers in metastatic urothelial carcinoma
Source: Front Immunol. 2025 Nov 20;16:1607222. doi: 10.3389/fimmu.2025.1607222 (PMC12675356; doi:10.3389/fimmu.2025.1607222)
Supplement: Supplementary file 9 [file DataSheet1.pdf]

Details of the feature selection procedures

(1) To find DE genes between responders vs. non-responders, we used DESeq2 (22), which assumes the read counts follow negative binomial distributions; this assumption was checked by insignificance from the Kolmogorov and Smirnov test ($P > 0.10$, KS test). For genes that followed negative binomial distributions, we identified 1993 DE genes of IMvigor210 (false discovery rate (FDR) < 0.10, (24); DEseq2 on responders versus non-responders).

(2) Next, read counts were normalized to $\log_2$(TPM+1), and all DE genes were ranked by their BW-ratios, which indicated how well they separate responders from non-responders to a drug (11, 23). The BW-ratio for a gene j of subject i (x_{ij}) in group k, and k = 1 (responders) and 2 (non-responders), is defined as follows.

$$\mathrm{BW(j)} = \frac{\sum_{\mathrm{i}} \sum_k I(y_i=k)(\bar{x}_{kj}-\bar{x}_{.j})^2}{\sum_{\mathrm{i}} \sum_k I(y_i=k)(x_{ij}-\bar{x}_{kj})^2},$$

where $\bar{x}_{.j}$ and $\bar{x}_{kj}$ denote the average expression level of gene j across all subjects and across the subjects of group k only, respectively. We then sorted the DE genes by their BW ratios in this step.

(3) Finally, given the training dataset (from a phase II clinical trial) and the test dataset (from a phase I clinical trial) were similar, we applied an unsupervised domain adaptation (DA) procedure to ensure that the distribution of each selected gene (feature), denoted as X_i, is similar across the two domains—that is, $P_S(X_i) \approx P_T(X_i)$, where S and T represent the source (training) and target (test) domains, respectively. The similarity of the distributions is measured by the p-values of Kolmogorov-Smirnov (KS) test. Genes with $P \geq \alpha_{DA}$ are chosen to train the predictor, where α_{DA} in [0.2, 0.8] (step size = 0.1).
Given there were 1930 DE genes in IMvigor210, we selected the top-500 ranked genes for further study.

Training four classifiers by 5-fold CV to yield the four predictors

After feature selection procedures, we utilized the training set IMvigor210 to train four classifiers, namely logistic regression, lasso regression, random forest (RF) and

SVM with the RBF kernel (SVM(RBF)), via 5-fold cross-validation (CV) and 100 repeats to result in the associated predictors.

Specifically, for LogitDA, given a fixed top-ranked p genes, where p is in the union of [15(5)200]) and [210(10)500] for IMvigor210 and α_{DA} in [0.2(0.1)0.8], we trained the hyperparameter λ (the penalty constant of logit ridge regression), p and α_{DA} using 5-fold CV with 100 repeats simultaneously. We used equal spaced grid-search to tune the hyper-parameter p and α_{DA}, and employed a two-stage optimization (Yuan et al., 2023) to determine λ as follows. The scoring function is the average of the area under the receiver operating characteristic curve (AUC) in all folds. First, let $\lambda = 10^{a_0}$ and $a_0 \in [-3, 0]$ with step size $10^{0.3}$, i.e., we ran 5-fold CV of LogitDA with grid points 10^{-3}, $10^{-2.7}$, … , and 10^0, and found the grid point whose associated CV score was the maximum, which was termed 10^{a_1},, e.g., $a_1 = -2.7$. Second, we further evaluated the logit ridge model (LogitDA) with grid points in $[10^{a_1-0.05}, 10^{a_1+0.05}]$ with step size $10^{0.01}$. The grid point $10^{a_{max}}$ whose corresponding CV score is the maximum
determines the tuned hyperparameter $\lambda = 10^{a_{max}}$. The logit model with the highest averaged CV AUC determined p and λ, which yielded one LogitDA. Finally, all training data was used to fit LogitDA with the optimized hyperparameters to yield the LogitDA predictor. For the other classifiers, lasso was optimized similar to LogitDA, while for SVM(RBF) and random forest, we used R packages and the optimization therein.
